# Propolis from the Monte Region in Argentina: A Potential Phytotherapic and Food Functional Ingredient

**DOI:** 10.3390/metabo11020076

**Published:** 2021-01-28

**Authors:** Iris Catiana Zampini, Ana Lia Salas, Luis M. Maldonado, Mario J. Simirgiotis, María Inés Isla

**Affiliations:** 1 Instituto de Bioprospección y Fisiología Vegetal (INBIOFIV, CONICET-UNT), San Miguel de Tucumán 4000, Argentina; zampini@csnat.unt.edu.ar (I.C.Z.); anasalas84@hotmail.com (A.L.S.); 2 Facultad de Ciencias Naturales, Universidad Nacional de Tucumán, San Lorenzo 1469, San Miguel de Tucumán 4000, Argentina; 3 Instituto Nacional de Tecnología Agropecuaria (INTA), Estación Experimental Agropecuaria Famaillá, Ruta Provincial 301–km 32, Famaillá, San Miguel de Tucumán 4000, Argentina; maldonado.luismaria@inta.gob.ar; 4 Instituto de Farmacia, Facultad de Ciencias, Universidad Austral de Chile, Valdivia 5090000, Chile; mario.simirgiotis@uach.cl

**Keywords:** Argentine propolis, *Zuccagnia punctata*, chalcones, metabolic syndrome, free radical scavenging activity, antimicrobial and nematicidal, chalcones

## Abstract

The aim of this review is to provide overall information on Argentine propolis and to shed light on its potential, especially the one from the Monte region so as to support future research in the field. Around 1999, the Argentine propolis began to be chemically and functionally characterized to give it greater added value. Because Argentina has a wide plant biodiversity, it is expected that its propolis will have various botanical origins, and consequently, a different chemical composition. To date, five types have been defined. Based on their functionality, several products have been developed for use in human and veterinary medicine and in animal and human food. Because the Argentine propolis with the greatest potential is that of the Monte eco-region, this review will describe the findings of the last 20 years on this propolis, its botanical source (*Zuccagnia punctata* Cav.), its chemical composition, and a description of markers of chemical quality (chalcones) and functionality. Propolis can regulate the activity of various pro-inflammatory enzymes and carbohydrate and lipid metabolism enzymes, as well as remove reactive oxygen and nitrogen species. Consequently, it can modulate metabolic syndrome and could be used as a functional ingredient in food. Furthermore, hydroalcoholic extracts can act against human and animal pathogenic bacteria and human yeast, and mycelial pathogenic fungi. The ability to stop the growth of post-harvest pathogenic bacteria and fungi was also demonstrated. For this reason, Argentine propolis are natural products capable of protecting crops and increasing the lifespan of harvested fruit and vegetables. Several reports indicate the potential of Argentine propolis to be used in innovative products to improve health, food preservation, and packaging. However, there is still much to learn about these natural products to make a wholesome use of them.

## 1. Introduction

Propolis (bee glue) is a natural product produced by *Apis mellifera* from resins collected from different parts of plants, namely, buds, young leaves, stems, and cracks in the bark, by mixing with wax and saliva. Bees use it to block cracks and to cover the internal walls of the hive as a defense system against microbial infections, parasites, and insects [1].

Propolis was widely used as phytomedicine for its anti-inflammatory, immunomodulatory, antioxidant, antimicrobial, antiparasitic, antiviral, antiaging, anesthetic, cytotoxic, antitumoral, hypolipidemic, and hypoglycemic activity, among others [2,3,4,5,6,7,8,9,10,11,12,13,14,15,16,17,18,19,20]. The antimicrobial and antioxidant properties are valuable in the food industry because of its positive effect on food-product stability and shelf life. Propolis has a potential as a natural food additive and functional food ingredient [21,22,23,24,25,26]. By the beginning of this century, Marcucci [3] and Bankova et al. [27] reported more than 300 constituents in propolis, and in the last twenty years, at least 550 new compounds have been isolated from it [28]. The vegetation around the hives and the preference of honeybees toward specific available botanical sources, determines its chemical diversity; hence, different propolis types exist. In a given geographic region, bees usually show preference for one or two plants. Although it is clear that they select specific sources, the cues used for finding a resin source are virtually unknown [1]. Some studies suggest common plant sources and similar chemical profiles for large geographical areas. Propolis from temperate regions in North America, Europe, and non-tropical regions of Asia derive from poplars (*Populus* spp.) and birches (*Betula* spp.) [27,28,29,30,31]. *Clusia minor* was described as the plant source of Venezuela propolis [32]. A brown propolis from *Clusia rosea* resin and a red propolis from *Dalbergia* spp. were described in Cuba [33]. The most popular propolis from Brazil are both green or alecrim propolis, originating from *Baccharis dracunculifolia*, and red propolis, originating from *Dalbergia ecastaphyllum*. Propolis of Brazil also come from *Hyptis divaricata* and *Populus nigra* [29]. According to Koenig (1995) [34] and Montenegro et al. (2001) [35], the most frequent botanical sources of propolis from central Chile are *Salix humboldtiana* and *Eucalyptus globulus*.

The aim of this review is to provide overall information on Argentine propolis and to shed light on their potential, placing special emphasis on propolis from the Monte region in order to both promote their use and support future research.

## 2. Research on Argentine Propolis

Beekeeping is one of the main activities in Argentine economy. Around 1999, the Argentine propolis began to be chemically and functionally characterized to enhance its value. Because Argentina has a wide plant biodiversity with several phytogeographical regions (Monte region, Gran Chaco region, Puna and Prepuna regions, and Yunga region), it is expected that its propolis will have various botanical origins and, consequently, a different chemical composition according to the place where the hives are placed. Because of the chemical variability of propolis, the study was not limited to a single specimen in each area. In addition, the studies on biological activities were also performed by using different experimental models and samples from different years. The propolis was classified according to collection sites in (a) propolis from Northwestern Argentina, (b) propolis from northeastern Argentina, (c) propolis from Cuyo or Andean region, (d) propolis from the central region, and (e) propolis from southern Argentina [36]. The chemical composition of northwestern Argentine propolis (Provinces of Tucumán, Santiago del Estero, Salta, Chaco, and Catamarca) was reported for the first time in 2005 [37]. Since then, more than 60 chemical components in propolis from this region have been identified [37,38,39,40,41,42,43,44,45,46,47,48], with the propolis from the Monte region being the most widely studied. Thirteen propolis components from the northeast (Provinces of Chaco and Misiones) were recorded [37]; whereas around eleven components were detected in the Cuyo region (Provinces of San Juan and Mendoza) [49,50,51,52]; five were identified in the central region, in Santa Fe [53]; ten phenolic compounds in propolis from Buenos Aires [54]; three in La Pampa; and thirteen in Entre Rios [52], Table 1. The chemical components of propolis from the south of the country were also studied, specifically those from Rio Negro, where eleven components were found [52], Table 1. Various propolis types have also been reported in Argentina, depending on their botanical origin. The species *Larrea nitida* and *Baccharis* are sources of propolis from the Andean region [55,56] and *Zuccagnia punctata* is a source of propolis from the Monte ecoregion in northwestern Argentina [38,40,44,45,46,47,48,57,58,59,60]. The Argentine propolis has several functional properties such as antibacterial, antifungal, anti-inflammatory, antioxidant, nematicidic, and cytotoxic, among others, apart from being an inhibitor of enzymes linked to metabolic syndrome [36,37,38,39,40,41,42,43,44,45,46,47,48,49,50,51,52,53,54,55,56,57,58,61,62,63,64]. Based on their properties, several products have been developed to date for use in human and veterinary medicine and in the food industry. Furthermore, Argentina has made progress in terms of quality control regulations of crude propolis and propolis extracts (IRAM-INTA15935-1 and -2 normative), and the propolis was included in the Argentine Food Code as a dietary supplement in May 2008 [26].

### 2.1. Propolis from the Monte Region in Argentina

**Figure 1:**
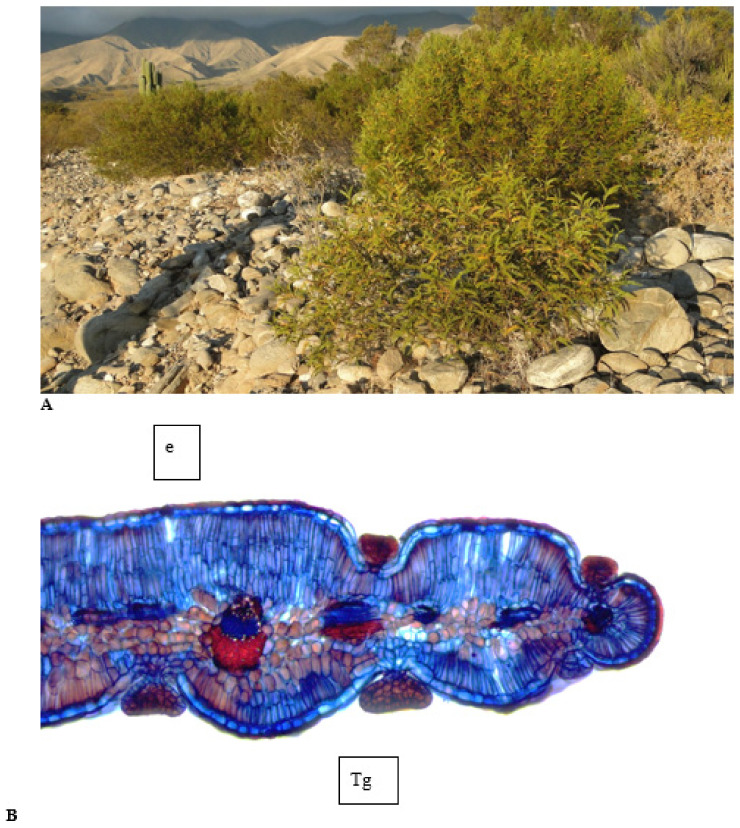
(**A**) Jarillal in the Monte region, (**B**) *Zuccagnia punctata.* Leaflet anatomy. Adaxial epidermis. Abaxial epidermis. Tg, capitate glandular trichome; e, epidermis.

The Monte ecoregion is exclusive to Argentina. It extends from the province of Jujuy (Quebrada de Humahuaca) to the northeast of Chubut. The Monte region in northern Argentina is a temperate and arid zone, where the predominant vegetation is xerophytic and halophytic shrub-steppe. The climax community of the Monte is the “jarillal”, an association of *Zuccagnia punctata*, *Larrea divaricata*, and *Larrea cuneifolia* (Figure 1).

#### 2.1.1. Chemical Characterization of Propolis from the Monte Region

Phenolic acid, flavone, flavanone, and chalcones were isolated and identified in propolis from the Monte region by using different technologies (Table 2). H NMR and UV spectra were used to isolate and identify 12 phenolic compounds in propolis from Catamarca in the Monte region, namely, flavanone (7-hydroxy-8-methoxyflavanone; 7,4′-dihydroxy-5-methoxyflavanone; 3β, 7-dihydroxy-5-methoxyflavanone; 7-dihydroxy-5,8-dimethoxyflavanone), flavones (4′, 5-dihydroxy-3,7,8-trimethoxyflavone; 5-hydroxy-4′,7-dimethoxyflavone; 3,7-dihydroxy-8-methoxyflavone; 3,5-dihydroxy-7,8-dimethoxyflavone; 7-hydroxy-5,8 dimethoxyflavone), and chalcones (2′,4′-dihydroxychalcone; 2′,4′-dihydroxy-3′-methoxychalcone; 2′,4′,4-trihydroxy-6′-methoxychalcone) [41]. UV-spectrum, mass spectra and fragmentation patterns were used to identify nine compounds in propolis samples from another site in Catamarca in the Monte region, namely, flavanone (3,5,7-trihydroxyflavanone (pinobanksin); 7-hydroxyflavanone; 5-hydroxy 7-methoxyflavanone; 7-hydroxy 8-methoxyflavanone), flavone (3,7-dihydroxy 8-methoxyflavone; 5,7-dihydroxyflavona (Chrysin); 3,5-dihydroxy 7,8-dimethoxy-flavone), and chalcones (2′,4′-dihydroxychalcone and 2′,4′-dihydroxy-3′-methoxychalcone) [45]. Various phenolic compounds were identified in propolis from the Monte region in Tucuman, namely, phenolic acids and their ester (cinnamic acid; caffeic acid prenyl ester; caffeoyl dihydrocaffeate; 3,4-dihydroxy-β-phenylethyl caffeate or teucrol; 1-methyl-3-(4-hydroxyphenyl)-propyl caffeic acid; 1-methyl-3-(3′,4′-dihydroxyphenyl)-propyl caffeic acid ester; 1-methyl-3-(4 -hydroxyphenyl)-propyl p-coumaric acid ester; 4′-terbutyloxyphenyl p-coumaric acid ester; 1-methyl-3-(4′-hydroxyphenyl)-propyl p-coumaric acid ester; 3,7-dimethyl-2,6-octadienyl caffeic acid ester (geranyl caffeate); 1-methyl-3-(3′,4′-dihydroxyphenyl)-propyl ferulic acid ester; 2-methyl-3-(3′-hydroxy-4′-methoxyphenyl)-propyl caffeic acid ester; flavanones (7-hydroxyflavanone and 7-hydroxy-8-methoxyflavanone; 7,8-dihydroxyflavanone; 3,7-dihydroxyflavanone; pinobanksin-5-methyl ether (3,7-dihydroxy-5-methoxyflavanone); 4′,7-dihydroxyflavanone (liquiritigenin); 5,7-dihydroxyflavanone (pinocembrin); 5-hydroxy-7-methoxyflavanone (pinostrobin)); flavones such as 7-O-methylgalangin (izalpinin); 3,5,7 trihydroxyflavone (galangin); 3,4′,5-trihydroxy-7-methoxyflavone (rhamnocitrin); and 3-hydroxy-7,8-dimethoxyflavone; and two chalcones named as 2′,4′-dihydroxychalcone and 2′,4′-dihydroxy-3′-methoxychalcone [40,44,46,47]. The two latter compounds were identified for the first time in Argentine propolis, and they were considered as chemical markers of propolis samples previously analyzed from the Monte region [38,40,44]. Then, two dihydrochalcones (4′-hydroxy-2′-methoxydihydrochalcone and 2′,4′-dihydroxydihydrochalcone) were also identified [47]. Chalcones are not very common compounds in propolis in other parts of the world. They were only identified in red propolis obtained from hives in the northern region of Brazil [65] and in propolis from apiaries located in the central southern region of Kangaroo Island [66].

Solorzano et al. (2019) [47] reported also minor compounds such as geranyl, pentenyl, and benzyl caffeate and cinnamyl caffeate. The major volatile compounds reported in *Zuccagnia*-type propolis was trans-linalool oxide (furanoid), cis-linalool oxide (furanoid), linalool, chrysanthenone, p-cymen-8-ol, and 2,3,6-trimethylbenzaldehyde *p*-mentha-1,5-dien-8-ol, (*E*)-anethole, α-terpineol, and *cis*-linalyl oxide (pyranoid) [48].

#### 2.1.2. Botanical Origin by Microscopic Analyses and Chemical Analysis

Determining the plant source is most important as it can help beekeepers select the place to place the hives so as to increase the production of propolis and achieve its standardization. The botanical source of propolis from the Monte region was determined by two methods, one by microscopic analysis of a propolis sample in order to identify fragments of leaves [57,58] of plant species that grow in this region. *Z. punctata, L. divaricata* and *L. cuneifolia* are the most abundant species in the Monte region (Figure 1A). The former was identified by the presence of compound leaf primordia with sub-opposite nanophyll leaflets with acuminate apex, rounded, symmetrical base and entire margin, free leaflet fragments with spherical to oval sunken capitate multicellular glandular trichomes and unicellular non-glandular trichomes arranged on the adaxial base of the foliar surface and on the foliar margins, leaflet epidermal cells with straight anticlinal walls, cyclocytic stoma, rarely paracytic or anomocytic on both epidermal surfaces (Figure 1B). These features respond to those described for *Z. punctata* in previous work [59,60]. Microscopic analyses of propolis samples revealed the presence of *Z. punctata* leaf as a major plant species.

Although *Larrea* leaves [56] were not found, pollen of different plants from the region was found, including pollen from *Zuccagnia* and *Larrea* [58]. To confirm the botanical origin of propolis samples coming from the ecoregion of Monte of Sierras and Bolsones, a chemical characterization by HPLC-DAD of resin extracts from three jarilla species (*Z. punctata, L. divaricata,* and *L. cuneifolia*) was carried out and compared with propolis extracts [40,57,58]. Major chemical components of the propolis and *Z. punctata* resin were 2′,4′-dihydroxychalcone (DHC) and 2′,4′-dihydroxy-3′-methoxychalcone (DHMC) and were considered as chemical markers of *Zuccagnia*-type propolis samples [44]. Chemical components, such as nordihydroguaiaretic acid, the major chemical compound of both *Larrea* species, were not found in the propolis extracts.

According to the chemical results, the botanical origin of propolis samples from the Monte region could be *Z. punctata*. Then, by using a liquid chromatography–diode array detector–quadrupole time-of-flight system (LC-DAD-QTOF), Solorzano et al. (2017) [46] identified some chemical components of the Monte region propolis and *Z. punctata* extracts, such as flavanones (7-hydroxyflavanone (HF) and 7-hydroxy-8-methoxyflavanone; 7,8-dihydroxyflavanone; 3,7-dihydroxyflavanone (DHF); pinobanksin-5-methyl ether (3,7-dihydroxy-5-methoxyflavanone); 3,7,8-trihydroxy dihydroflavanone); chalcones (2′,4′-dihydroxychalcone and 2′,4′-dihydroxy-3′-methoxychalcone), which were previously reported; and two new dihydrochalcones (4′-hydroxy-2′-methoxy dihydrochalcone and 2′,4′-dihydroxydihydrochalcone) and phenolic acid and esters: 1-methyl-3-(4-hydroxyphenyl)-propyl caffeic acid; 1-methyl-3-(3′,4′-dihydroxyphenyl)-propyl caffeic acid ester; 1-methyl-3-(4-hydroxyphenyl)-propyl p-coumaric acid ester; 4′-terbutyloxyphenyl p-coumaric acid ester; 1-methyl-3-(4′-hydroxyphenyl)-propyl p-coumaric acid ester; 3,7-dimethyl-2,6-octadienyl caffeic acid ester (geranyl caffeate); 1-methyl-3-(3′,4′-dihydroxyphenyl)-propyl ferulic acid ester; 2-methyl-3-(3′-hydroxy-4′-methoxyphenyl)-propyl caffeic acid ester, see Table 2.

#### 2.1.3. Pharmacological Activities

**Figure 2:**
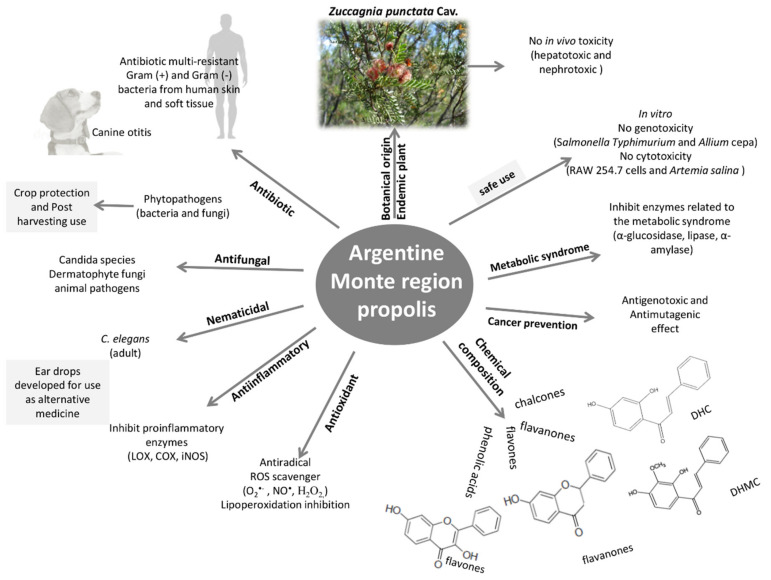
Functional properties of propolis from the Monte region.

Argentine propolis from the Monte ecoregion has multiple pharmacological properties (Figure 2), which have been reported in the last 20 years. These properties have made significant progress.

##### 2.1.3.1. Antibacterial Activity

The prevalence of multidrug resistant bacteria versus commercial antibiotics has left healthcare systems with few treatment options, which are generally expensive therapies. In recent years, alternative and more specific antimicrobials that complement conventional therapy have been studied. Phenolic compounds of plant origin exhibit antibacterial activity by mechanisms different from conventional drugs, thus rendering bacteria unable to develop resistance [67]. Propolis from hives in the Monte region showed antibacterial activity against antibiotic resistant Gram-positive and Gram-negative bacteria isolated from skin and soft tissue infections [41,43,44,45,68]. These propolis showed greater activity against Gram-positive bacteria than against Gram-negative bacteria with minimal inhibitory concentration (MIC) values between 10 and 100 µg/mL and between 400 and 1600 µg/mL, respectively.

The propolis also showed antibacterial activity against microorganism isolates from canine otitis such as *Staphylococcus haemolyticus, S. aureus,* and *S. intermedius* with MIC values between 75 and 150 μg/mL, and minimal bactericidal concentration (MBC) values between of 200 and 600 μg/mL. According to the results of the in situ bioautographic tests, DHC and DHMC would be responsible for the inhibition of the growth of Gram-positive bacteria, principally *Staphylococcus aureus* isolated from human and animal infections [43,45,62].

The antibacterial activity exerted by the propolis extracts against common human and animal pathogenic strains suggests their potential application in the treatment of infectious processes.

##### 2.1.3.2. Antifungal Effect

Fungal infections are very difficult to treat and long-term treatments with commercial antifungal products have side effects; therefore, it is necessary to avoid adverse effects. *Zuccagnia*-type propolis extracts were inhibitors of the growth of dermatophytes (*Microsporum gypseum, Trichophyton mentagrophytes,* and *Trichophyton rubrum*) with MIC values between 16 and 125 μg/mL [40]. The main identified antifungal compounds were two chalcones, DHC and DHMC with MIC and minimal fungicidal concentration (MFC) values between 1.9 and 2.9 μg/mL.

The anti-*Candida* activity was also demonstrated (MIC of 125–500 μg/mL and MFC of 375–750 μg/mL) [44]. The anti-*Candida* activity of propolis was similar to that of dry extracts of *Z. punctata* [69] and could be attributed to DHC and DHMC [69,70]. Both chalcones could moderate fungal colonization and suppress the invasive mechanism of *Candida*, for example, by acting as an inhibitor of germ tube formation as well as biofilm formation and acting on exoenzyme activity [69]. According to the MIC obtained and considering the Tangarife-Castaño et al. classification, the extracts of the *Zuccagnia*-type propolis can be considered strong antifungals [71].

##### 2.1.3.3. Nematicidal Activities

The *Zuccagnia*-type propolis showed an effect on adult *Caenorhabditis elegans* [44]. The LC_50_, defined as the concentration required for killing half of the *C. elegans* population within 24 h, was 70 μg/mL close to what the drug levamisole, a known anthelmintic drug (LC_50_ 4.7 μg/mL), required.

##### 2.1.3.4. Antioxidant Capacity

The reactive species of oxygen and nitrogen such as hydroxyl radical (HO^•^), hydrogen peroxide (H_2_O_2_), superoxide radical (O_2_^•-^), and nitric oxide (NO^•^) are a health hazard, since they can oxidize proteins, sugars, nucleic acids, and lipids. These free radicals contribute to several pathologies associated with oxidative stress such as inflammatory process, carcinogenesis, and metabolic syndrome.

*Zuccagnia*-type propolis extracts from Catamarca were active as HO^•^ scavengers (Free radicals half scavenging concentration, SC_50_ values around 25 ± 5 µg/mL), showed high potential as an H_2_O_2_ scavenger and as O_2_^•-^ scavengers with SC_50_ values of 115 and 205 µg/mL, and showed a remarkable capacity to scavenge nitrogen-reactive species such as NO^•^ [58].

*Zuccagnia*-type propolis from Catamarca and Tucuman showed scavenging activity on ABTS^•+^ with SC_50_ values between 14 and 33 µg/mL [44,58], similar to the antioxidant capacity of *Z. punctata* extracts [72,73,74,75]. Other authors also evidenced the free-radical-scavenging activity of Monte region propolis extract on DPPH^•^ (SC_50_ values between 10 and 43 µg/mL) [41,45,63,64].

The *Zuccagnia*-type propolis protected lipids from oxidation (inhibitory concentration of lipid oxidation in 50%, IC_50_ between 2 and 29 μg/mL) [44,64]. Avila et al. and Morán Vieyra et al. [76,77] reported the antioxidant properties and mechanisms of three structurally related flavonoids present in *Zuccagnia*-type propolis, 7-HF, DHC, and 3,7-DHF. The ABTS^•+^ and DPPH^•^ scavenging reactivity trend was DHF > DHC > HF, which correlated with the electron donor capacity of the flavonoids. However, the O_2_ scavenging in aqueous buffered solution was significantly controlled by the fraction of neutral flavonoids through concerted proton-coupled electron transfer. The radical-scavenging reactivity trend was DHC > DHF > HF.

##### 2.1.3.5. Effects on Pro-Inflammatory Mediators

The arachidonic acid (AA) pathway is involved in the inflammatory reactions. The best treatment for the inflammatory process would be to inhibit the pathway at several levels [78], for instance, phospholipase (PLA2), cyclooxygenases (COX2), and lipoxygenases (LOX), reducing the concentrations of prostanoids and leukotrienes. Specific inhibitors of the proinflammatory enzymes have side effects that prevent their consumption for long periods of time [79]. Several phenolic compounds were reported as inhibitors of COX2 and LOX [80,81]. In the present review, the findings of anti-inflammatory activity research of propolis from the Monte region have been summarized. The bioassays are focused on two most important topics: the effect on AA metabolizing enzymes and NOS, and the effect on the expression of proinflammatory enzymes.

The *Zuccagnia*-type propolis were effective as LOX activity inhibitors, with IC_50_ values of 70 ± 10 μg/mL), and COX inhibitors, with IC_50_ values around 100 ± 4 μg/mL [44]. These results are very promising; they inhibit LOX in percentages and concentrations similar to commercial anti-inflammatories, though at two levels of the AA pathway. The effect of a major component, 2′,4′ dihydroxy chalcone, present in propolis extract on COX2, was assayed. DHC was a potent inhibitor on COX2 and showed a dose-dependent response between 4 and 190 μM [82]. The inhibition of pro-inflammatory enzymes by DHC and its antioxidant capacity support the potential use of propolis as a medicine, which could be used to prevent the development of chronic inflammatory pathologies. Cell stimulation with bacterial lipopolysaccharide (LPS) induces pro-inflammatory cytokine production and iNOS protein expression in macrophages; consequently, it caused significant increase in NO production. Pretreatment of cells with *Zuccagnia*-type propolis extract inhibited NO overproduction in a dose-dependent manner (IC_50_ around 10 ± 0.5 μg/mL) and the iNOS expression with IC_50_ values around 30 μg/mL, while the COX2 expression was not affected [44]. This is the only report on anti-inflammatory activity for Argentine propolis; this activity was also reported to Brazilian, Chilean, Korean and Chinese propolis through other mechanisms [83].

##### 2.1.3.6. Inhibitory Capacity of Enzymes Related to Metabolic Syndrome

Metabolic syndrome (MetS) is a clinical state that gathers several metabolic risk factors, including central obesity, i.e., excess of visceral adiposity, insulin resistance, hyperglycemia, hypertension, or dyslipidemia, characterized by high triglycerides level. Therapeutics for MetS are mainly based on lifestyle changes, often accompanied by pharmacological treatments. The modulation of inflammatory process and oxidative status in MetS is necessary to control this disorder, as well as the inhibition of enzymes involved in sugar and lipid metabolism [84]. *Zuccagnia*-type propolis extract showed strong antioxidant and anti-inflammatory activity (see Section 2.1.3.4 and Section 2.1.3.5), which can help reduce oxidative stress in MetS. It also showed inhibitory activity for α-glucosidase and lipase, followed by α-amylase [58]. Two chalcones, DHC and DHMC, chemical markers of *Zuccagnia*-type propolis and *Z. punctata* resin extract, were active, inhibiting lipase and α-glucosidase enzymes. Other authors reported that chalcones are potent α-glucosidase, α-amylase, and lipase inhibitors [85,86,87,88]. Oral administration of *Z. punctata* extract (plant source of *Zuccagnia*-type propolis) improves lipidic profile, reduces oxidative process and avoids vascular dysfunction in hypercholesterolemic rabbits [89,90].

#### 2.1.4. Crop Protection and Post Harvesting Use

##### Antibacterial Activity

The *Zuccagnia*-type propolis were also active on phytopathogenic bacteria (*Pseudomonas syringae, Pseudomonas corrugata, Xanthomonas campestris,* and *Erwinia carotovora*), which are serious problems regarding crops or processed and fresh market tomatoes (*Lycopersicon esculentum* L.) [91] with MIC values between 5 and 40 μg gallic acid equivalent per milliliter GAE/mL. The main isolated antibacterial compound from this propolis was identified as DHC. Propolis-aqueous solutions, sprayed on tomato fruits, reduce the symptoms of disease. The effect of a propolis solution sprayed prior to or after inoculation with *P. syringae* pvar. tomato was similar, showing both curative and preventive effects. The bactericidal effect of propolis in vivo leads us to consider potential uses in agribusiness.

##### Antifungal Activity

The antifungal activity of the Zuccagnia-type propolis and the films containing ethanolic extract of this propolis against Penicillium digitatum, Penicillium expansum, Penicillium italicum, Alternaria alternata, Aspergillus carbonarius and Botrytis cinerea was assayed [92]. All the tested fungal pathogens were sensitive toward the propolis extract. The most striking effect could be seen on the growth of P. digitatum and B. cinerea, with MIC values of 0.14 and 0.17 mg/mL, respectively followed. A moderate effect was observed for Alternaria alternata, P. italicum, and A. carbonarius, with MIC values of around 0.40 mg/mL. The lowest sensitivity was exhibited by P. expansum (MIC values of 0.58 mg/mL). The sporulation of all fungal pathogens tested was affected by using 0.05 mg/mL of propolis extract [92].

#### 2.1.5. Toxicity of Propolis from the Monte Region

Propolis did not show toxicity to model organisms such as *Artemia salina* and *Allium cepa* at concentrations where they showed biological activities. Genotoxicity was not found against the *Salmonella typhimurium* strains with and without metabolic activator, but they could inhibit the mutagenesis produced by two mutagens, isoquinoline and 4-nitro o-phenylenediamine. Similar results were reported for DHC, the main component of propolis from the Monte region in Argentina, evincing the potential of these bee products as anticancer agents [38]. Propolis extracts were not toxic against RAW 254.7 cell lines [44].

No toxicity studies were performed for *Zuccagnia*-type propolis extracts on experimentation animals; however, they were done for extracts of aerial parts from *Zuccagnia punctata*, from whose resins this propolis derives. The toxic effect on the liver and kidney of *Z. punctata* extract (1 mg/mice) was analyzed in mice. The activities of alanine transaminase and aspartate transaminase hepatic enzymes, as well as the levels of creatinine and urea in blood, were not changed with the administration of *Z. punctata* extract as compared with that of the control mice. Therefore, the report showed that the intake once or twice a day of 1 mg of plant extract for seven days did not result in toxicity [93]. Oral administration of *Zuccagnia punctata* extract at a dose of 2.5 mg/day to a hypercholesterolemic rabbit was not toxic as regards the kidney, and both the hepatic function and the hematological parameters did not change in relation with rabbit controls [89,90].

#### 2.1.6. Fields of Application of Propolis from the Monte Region

Several reports indicate the great value of propolis to be used in the development of innovative products to improve health, functional food, food preservation, food packaging, and textile materials for biomedical application, to name but a few [6,94,95]. Several products containing propolis, such as medical devices, health foods, beverages, and cosmetics, among others, have been developed and commercialized. This is largely due to the numerous beneficial pharmacological properties of bee glue, i.e., its anti-inflammatory, anti-obesity, antitumor, antimicrobial, and antioxidant properties. A pharmaceutical product, ear drops containing *Zuccagnia*-type propolis extract as a bioactive, was developed for use in otitis [62]. The ear drops were standardized chemically, functionally, and microbiologically. The formulation showed inhibitory activity on pro-inflammatory enzymes, such as LOX (IC_50_ values 90 and 100 μg/mL), free-radical-scavenging effect (SC_50_ values 23 and 30 μg/mL), anthelmintic (LC_50_ values 70 and 71 μg/mL), anti-*Candida* (400 μg/mL), and antimicrobial activity against Gram-positive bacteria (200 μg/mL) during six month-storage. The content of chalcones, chemical markers of *Zuccagnia*-type propolis was quantified (DHC 3.54 mg/mL and DHMC 4.54 mg/mL) and its level was kept while stored at room temperature. The ear drops were not toxic. The results are noteworthy since the *Zuccagnia*-type propolis extract and ear drops developed could be an option for use in alternative medicine as an antibacterial, anti-*Candida*, anthelmintic, anti-inflammatory, and antioxidant.

In the last few years, propolis extracts were used in the conservation of food during storage. The propolis extracts can be added directly to foods or are administered superficially or in the form of edible films enriched in propolis extracts. These procedures reduce or eliminate pathogens or saprophytic microorganism from fish, fruit, vegetables, fruit juice, and milk. The propolis can contribute to keeping the quality of fruit, vegetables, meat, and fish during storage [24].

Edible, gelatin-based films containing *Zuccagnia*-type propolis were recently developed. They revealed remarkable antifungal activity against *P. digitatum* and *B. cinerea* and reduced the incidence of infection in raspberries stored at refrigerated temperatures for a long period of time [93].

## 3. Concluding Remarks and Future Trends

In this review, the wide potential of Argentine propolis is shown, especially the one whose botanical origin is *Zuccagnia punctata*, an endemic medicinal species of Argentina with unique characteristics. Additionally, substantial work has been done not only in determining its functional properties but also in its standardization from the chemical perspective. However, it is necessary to deepen the knowledge of chemical and functional properties, as well as the development of pharmaceutical, food, and cosmetic products derived from Argentine propolis.

## 4. Materials and Methods

This biographic research started in 1970 and ended at the end of November 2020; electronic databases such as http://www.scopus.com; http://www.scirus.com; http://scholar.google.com.ar; http://www.ncbi.nlm.nih.gov/pubmed; and http://www.sciencedirect.com were used. Searches were made by using key word combinations: propolis, *Zuccagnia*-type propolis, Argentine propolis, *Zuccagnia punctata*, *Larrea*, jarilla, biological activities, phytochemicals, toxicity, 2′,4′-dihydroxy chalcone, 2′,4′-dihydroxy-3′-methoxychalcone, antibacterial, antifungal, antioxidant, antibacterial, anti-inflammatory, chemo-preventive, antiulcer, and anti-biofilms, among others. The data were selected from systematic reviews and articles published in English. The bibliography was categorized according to its scope, namely botanical source, geographical origin, chemical composition, biological activity, or field of application. Data extraction was performed by all researchers, and the reported conclusions were achieved by consensus.

## Figures and Tables

**Table 1:** Chemical composition of Argentine propolis (province distribution).

Phenolic Components	SE	T	CH	S	J	C	RN	LP	ER	SJ	M	SF	BA
Phenolic acid and derivates													
Coumaric acid	X	X	X	X	X	X	X	ND	X		X	X	X
Caffeic acid	ND	ND	ND	ND	ND	ND	X	ND	ND		X	X	X
Ferulic acid	X	X	X	X	ND	X	X	ND	X		X	ND	X
Cinnamic acid	X	X	X	ND	ND	X	ND	X	X		ND	ND	ND
CAPE	X	X	X	ND	ND	X	X	ND	X		X	ND	ND
Flavanones													
Pinobanksin	X	X	X	ND	ND	X	X	ND	X	X	X	ND	ND
Pinocembrin	X	X	X	ND	ND	X	X	ND	X	X	X	ND	X
Pinocembrin derivate	ND	ND	ND	ND	ND	ND	ND	ND	ND	ND	ND	ND	X
Naringenin	ND	ND	ND	ND	ND	ND	ND	ND	ND	ND	ND	ND	X
7-hydroxy-8-methoxyflavanone	ND	ND	ND	ND	ND	ND	ND	ND	ND	X	ND	ND	ND
Flavone													
Apigenin	X	X	X	X	X	X	X	X	X		X	ND	X
Chrysin	X	X	X	ND	ND	X	X	X	X	X	X	X	X
Tectochrysin	X	X	X	ND	ND	X	X	ND	X	X	X	ND	ND
Flavonol													
Galangin	X	X	X	ND	ND	X	X	ND	X	X	X	X	X
Quercetin	X	X	X	ND	ND	X	ND	ND	X		ND	X	X
Kaempferol	X	X	X	ND	ND	X	ND	ND	X		ND	ND	ND
Kaempferide	X	X	X	ND	ND	X	X	ND	X		X	ND	ND
Lignans													
3′methyl-nordihydroguaiaretic acid (MNDGA)	ND	ND	ND	ND	ND	ND	ND	ND	ND	X	ND	ND	ND
nordihydroguaiaretic acid(NDGA)	ND	ND	ND	ND	ND	ND	ND	ND	ND	X	ND	ND	ND
3 (4-[4-(4-hydroxy-phenyl)-2,3-dimethyl-butyl]-benzene-1,2-diol)	ND	ND	ND	ND	ND	ND	ND	ND	ND	X	ND	ND	ND
meso-(rel 7S,8S,7′R,8′R)-3,4,3′,4′-tetrahydroxy7,7′-epoxylignan	ND	ND	ND	ND	ND	ND	ND	ND	ND	X	ND	ND	ND
5 (7S,8S,7′S,8′S)-3,3′,4′-trihy droxy-4-methoxy-7,7′-epoxylignan	ND	ND	ND	ND	ND	ND	ND	ND	ND	X	ND	ND	ND

SE: Santiago del Estero; T: Tucuman; C: Catamarca; CH: Chaco; J: Jujuy; S: Salta, RN: Rio Negro; SF: Santa Fe; LP: L Pampa; SJ: San Juan; M: Mendoza; ER: Entre Rios; BA: Buenos Aires; ND: non detected.

**Table 2:** Compounds occurring in propolis from the Argentine Monte region.

Compounds	Geographical Sites	References
Flavanones		
7-hydroxyflavanone	Tucumán, Catamarca	Agüero et al., 2010; Solorzano et al., 2012; Solórzano et al., 2017
7,8-dihydroxyflavanone	Catamarca. Tucumán	Solórzano et al., 2017
3,7-dihydroxyflavanone	Catamarca. Tucumán	Solórzano et al., 2017
4′,7-dihydroxyflavanone (liquiritigenin)	Tucumán	Salas et al., 2016a
5,7-dihydroxyflavanone (pinocembrin)	Catamarca, Tucumán	Agüero et al., 2010, Solórzano et al., 2017
3,5,7-trihydroxyflavanone (pinobanksin)	Catamarca	Solorzano et al., 2012
3,7,8-trihydroxydihydroflavanone	Catamarca, Tucumán	Solórzano et al., 2017
5-hydroxy- 7- methoxyflavanone (pinostrobin)	Tucumán, Catamarca	Agüero et al., 2010, Solorzano et al., 2012
7-hydroxy- 8-methoxyflavanone	Catamarca, Tucumán	Vera et al., 2011; Agüero et al., 2010; Solorzano et al., 2012; Solórzano et al., 2017
7,4′-dihydroxy-5-methoxyflavanone	Catamarca, Tucumán	Vera et al., 2011, Agüero et al., 2010; Solorzano et al., 2017
3β, 7-dihydroxy-5-methoxyflavanone	Catamarca	Vera et al., 2011
7-hydroxy-5,8-dimethoxyflavanone	Catamarca	Vera et al., 2011
pinobanksin-5-methyl ether (3,7-dihydroxy-5-methoxyflavanone)	Catamarca, Tucumán	Solórzano et al., 2017
Flavones		
5,7-dihydroxyflavone (chrysin)	Catamarca, Tucumán	Solorzano et al., 2012; Solórzano et al., 2017
3,7-dihydroxyflavone	Catamarca, Tucumán	Solórzano et al., 2017
3,5,7-trihydroxyflavone (galangin)	Catamarca, Tucumán	Agüero et al., 2010, Vera et al., 2011; Solórzano et al., 2017
3-hydroxy-7,8-dimethoxyflavone	Tucumán	Agüero et al., 2010, Vera et al., 2011
7-hydroxy-5,8 dimethoxyflavone	Catamarca	Vera et al., 2011
5-hydroxy-4′,7-dimethoxyflavone	Catamarca	Vera et al., 2011
3,7-dihydroxy-8-methoxyflavone	Catamarca, Tucumán	Vera et al., 2011; Solorzano et al., 2012, Solórzano et al., 2017
3,5-dihydroxy-7-methoxyflavone (izalpinin)	Tucumán	Agüero et al., 2010
3,5-dihydroxy-7,8-dimethoxyflavone	Catamarca, Tucumán	Vera et al., 2011; Solorzano et al., 2012; Solórzano et al., 2017
4′, 5-dihydroxy-3,7,8-trimethoxyflavone	Catamarca	Vera et al., 2011
3,4′,5-trihydroxy-7-methoxyflavone (rhamnocitrin)	Catamarca, Tucumán	Agüero et al., 2010; Solórzano et al., 2017
Chalcones		
2′,4′-dihydroxychalcone (DHC)	Catamarca, Tucumán	Vera et al., 2011; Agüero et al., 2010; Solorzano et al., 2012, Salas et al., 2016a,b; Solórzano et al., 2017
2′,4′-dihydroxydihydrochalcone	Catamarca, Tucumán	Solórzano et al., 2017
4′-hydroxy-2′-methoxydihydrochalcone	Catamarca, Tucumán	Solórzano et al., 2017
2′,4′-dihydroxy-3′-methoxychalcone (DHMC)	Catamarca, Tucumán	Vera et al., 2011; Agüero et al., 2010; Solorzano et al., 2012; Salas et al., 2016a,b; Solórzano et al., 2017
2′,4′,4-trihydroxy-6′-methoxychalcone	Catamarca, Tucumán	Vera et al., 2011, Solórzano et al., 2017
Phenolic acids and esters		
cinnamic acid	Tucumán	Salas et al., 2016b
1,1-dimethylallyl caffeic acid	Catamarca, Tucumán	Solórzano et al., 2017
caffeoyl dihydrocaffeate	Tucumán	Salas et al., 2016b
geranyl caffeate	Catamarca, Tucumán	Solórzano et al., 2019
pentenyl caffeate	Catamarca, Tucumán	Solórzano et al., 2019
benzyl caffeate	Catamarca, Tucumán	Solórzano et al., 2019
cinnamyl caffeate	Catamarca, Tucumán	Solórzano et al., 2019
methyl caffeate	Catamarca	Solórzano et al., 2019
caffeic acid prenyl ester	Tucumán	Salas et al., 2016b
3,4-dihydroxyphenethyl caffeic acid ester (teucrol)	Tucumán	Salas et al., 2016b
1-methyl-3-(4-hydroxyphenyl)-propyl caffeic acid ester	Catamarca	Solórzano et al., 2017
1-methyl-3-(4 -hydroxyphenyl)-propyl p-coumaric acid ester	Catamarca	Solórzano et al., 2017
1-methyl-3-(3′,4′-dihydroxyphenyl)-propyl caffeic acid ester	Catamarca	Solórzano et al., 2017
4′-terbutyloxyphenyl p-coumaric acid ester	Catamarca, Tucumán	Solórzano et al., 2017
1-methyl-3-(4′-hydroxyphenyl)-propyl p-coumaric acid ester	Catamarca, Tucumán	Solórzano et al., 2017
3,7-dimethyl-2,6-octadienyl caffeic acid ester (geranyl caffeate)	Catamarca, Tucumán	Solórzano et al., 2017
1-methyl-3-(3′,4′-dihydroxyphenyl)-propyl ferulic acid ester	Catamarca, Tucumán	Solórzano et al., 2017
2-methyl-3-(3′-hydroxy-4′-methoxyphenyl)-propyl caffeic acid ester	Catamarca, Tucumán	Solórzano et al., 2017
Volatile compounds		
trans-linalool oxide (furanoid)	Tucumán	Gonzalez et al., 2019
*cis*-linalool oxide (furanoid)	Tucumán	Gonzalez et al., 2019
*(E*)-anethole	Tucumán	Gonzalez et al., 2019
Linalool	Tucumán	Gonzalez et al., 2019
*cis*-linalyl oxide (pyranoid)	Tucumán	Gonzalez et al., 2019
p-cymen-8-ol	Tucumán	Gonzalez et al., 2019
2,3,6-trimethylbenzaldehyde	Tucumán	Gonzalez et al., 2019
Chrysanthenone	Tucumán	Gonzalez et al., 2019
*p*-mentha-1,5-dien-8-ol	Tucumán	Gonzalez et al., 2019

## References

[1] Bankova V., Popova M., Trusheva B. (2018). The phytochemistry of the honeybee. Phytochemistry.

[2] Ghisalberti E.L. (1979). Propolis: A review. Bee World.

[3] Marcucci M.C. (1995). Propolis—chemical-composition, biological properties and therapeutic activity. Apidologie.

[4] Burdock G.A. (1998). Review of the biological properties and toxicity of bee propolis (propolis). Food Chem. Toxicol..

[5] Banskota A.H., Tezuka Y., Kadota S. (2001). Recent progress in pharmacological research of propolis. Phytother. Res..

[6] Bankova V. (2005). Recent trends and important developments in propolis research. eCAM.

[7] Sforcin J.M. (2007). Propolis and the immune system: A review. J. Ethnopharmacol..

[8] Bankova V. (2009). Chemical diversity of propolis makes it a valuable source of new biologically active compounds. JAAS.

[9] Sforcin J.M., Bankova V. (2011). Propolis is there a potential for the development of new drugs?. J. Ethnopharmacol..

[10] Bankova V., Popova M., Trusheva B. (2014). Propolis volatile compounds: Chemical diversity and biological activity: A review. Chem. Cen. J..

[11] Anjum S.I., Ullah A., Khan K.A., Attaullah M., Khan H., Ali H., Bashir M.A., Tahir M., Ansari M.J., Ghramh H.A. (2019). Composition and functional properties of propolis (bee glue): A review. Saudi J. Biol. Sci..

[12] Almuhayawi M.S. (2020). Propolis: A Novel Antibacterial Agent. Saudi J. Biol. Sci..

[13] Jalali M., Ranjbar T., Mosallanezhad Z., Mahmoodi M., Moosavian S.P., Ferns G., Jalali Z., Sohrabi Z. (2020). Effect of Propolis supplementation on serum CRP and TNF-α levels in adults: A systematic review and meta-analysis of clinical trials. Complement. Ther. Med..

[14] Rojczyk E., Klama-Baryła A., Łabuś W., Wilemska-Kucharzewska K., Kucharzewski M. (2020). Historical and modern research on propolis and its application in wound healing and other fields of medicine and contributions by Polish studies. J. Ethnopharmacol..

[15] Šturm L., Poklar Ulrih N. (2020). Chapter Two—Propolis flavonoids and terpenes, and their interactions with model lipid membranes: A review. Advances in Biomembranes and Lipid Self-Assembly.

[16] Stavropoulou I., Stathopoulou K., Cheilari A., Benaki D., Gardikis K., Chinou I., Aligiannis N. (2021). NMR metabolic profiling of Greek propolis samples: Comparative evaluation of their phytochemical compositions and investigation of their anti-ageing and antioxidant properties. J. Pharm. Biomed..

[17] Silva M.P., Silva T.M., Mengarda A.C., Salvadori M.C., Teixeira F.S., Alencar S.M., Filho G.C.L., Bueno-Silva B., de Moraes J. (2020). Brazilian red propolis exhibits antiparasitic properties in vitro and reduces worm burden and egg production in a mouse model harboring either early or chronic Schistosoma mansoni infection. J. Ethnopharmacol..

[18] Laaroussi H., Bakour M., Ousaaid D., Aboulghazi A., Ferreira-Santos P., Genisheva Z., Teixeira J.A., Lyoussi B. (2020). Effect of antioxidant-rich propolis and bee pollen extracts against D-glucose induced Type 2 Diabetes in rats. Food Res. Int..

[19] Berretta A.A., Duarte Silveira M.A., Cóndor Capcha J.M., De Jong D. (2020). Propolis and its potential against SARS-CoV-2 infection mechanisms and COVID-19 disease. Biomed. Pharm..

[20] Rosseto H.C., de Toledo L.D.A.S., dos Santos R.S., de Francisco L.M.B., Vecchi C.F., Esposito E., Cortesi R., Bruschi M.L. (2020). Design of propolis-loaded film forming systems for topical administration: The effect of acrylic acid derivative polymers. J. Mol. Liq..

[21] Mendiola J.A., Martïn-Alvarez P.J., Señoráns F.J., Reglero G., Capodicasa A., Nazzaro F., Sada A., Cifuentes A., Elena Ibáñez E. (2010). Design of natural food antioxidant ingredients through a chemometric approach. J. Agric. Food Chem..

[22] Da Silva F.C., da Fonseca C.R., de Alencar S.M., Thomazini M., Balieiro J.C.D.D., Pittia P., Favaro-Trindade C.S. (2013). Assessment of production efficiency, physicochemical properties and storage stability of spray-dried propolis, a natural food additive, using gum Arabic and OSA starch-based carrier systems. Food Bioprod. Process..

[23] Yücel B., Topal E., Kosoglu M. (2017). Bee Products as Functional Food. Superfood and Functional Food—An Overview of Their Processing and Utilization.

[24] Pobiega K., Kraśniewska K., Gniewosz M. (2019). Application of propolis in antimicrobial and antioxidative protection of food quality—A review. Trends Food Sci. Technol..

[25] Sadhana N., Lohidasan S., Mahadik K. (2017). Marker-based standardization and investigation of nutraceutical potential of Indian propolis. J. Integr. Med..

[26] Argentine Food Code. 2009 Cap. XVII. Art. 1384. http://www.anmat.gov.ar/alimentos/codigoa/Capitulo_XVII.pdf.

[27] Bankova V., De Castro L., Marcucci M.C. (2000). Propolis recent advances in chemistry and plant origin. Apidologie.

[28] Šturm L., Poklar Ulrih N. (2020). Review Advances in the Propolis chemical composition between 2013 and 2018: A Review. eFood.

[29] Salatino A., Teixeira E.W., Negri G., Message D. (2005). Origin and chemical variation of Brazilian propolis. Evid. Based Complementary Altern. Med..

[30] Greenaway W., Scaysbrook T., Whately F.R. (1990). The composition and plant origin of propolis: A report of work at Oxford. Bee World.

[31] Lotti C., Campo Fernández M., Piccinelli A.L., Cuesta-Rubio O., Márquez Hernández I., Rastrelli L. (2010). Chemical constituents of red Mexican propolis. J. Agric. Food Chem..

[32] Tomas-Barberán F.A., Garcia-Viguera C., Vitolivier P., Ferreres F., Tomás-Lorente F. (1993). Phytochemical evidence for the botanical origin of tropical propolis from Venezuela. Phytochemistry.

[33] Cuesta-Rubio O., Piccinelli A.L., Campo Fernandez M., Marquez Hernandez I., Rosado A., Rastrelli L. (2007). Chemical characterization of Cuban propolis by HPLC-PDA, HPLC-MS, and NMR: The brown, red, and yellow cuban varieties of propolis. J. Agric. Food Chem..

[34] Koenig B. (1995). Plant sources of propolis. Bee World.

[35] Montenegro G., Peña R.C., Mujica A.M., Pizarro R. (2001). Botanical resources for propolis in an apiary network in central Chile. Phyton Int. J. Exp. Bot..

[36] Isla M.I., Nieva Moreno M.I., Zampini I.C., Solórzano E., Danert F., Vera N., Sayago J.E., Bedascarrabure E., Maldonado L., Ordoñez R., Farooqui T., Farooqui A. (2011). Argentine propolis: Its flavonoid and chalcone content and its relation with the functional properties. Beneficial Effects of Propolis on Human Health and Chronic Diseases.

[37] Isla M.I., Paredes Guzman J.F., Nieva Moreno M.I., Koo H., Park Y.K. (2005). Some chemical composition and biological activity of Northern Argentine propolis. Some chemical composition and biological activity of Northern Argentine propolis. J. Agric. Food Chem..

[38] Nieva Moreno M.I., Zampini I.C., Ordóñez M., Vattuone M.A., Isla M.I. (2005). Evaluation of the cytotoxicity, mutagenicity and antimutagenicity of propolis from Amaicha del Valle, Tucumán, Argentina. J. Agric. Food Chem..

[39] Chaillou L., Nazareno M. (2009). Bioactivity of propolis from Santiago del Estero, Argentina, related to their chemical composition LWT. Food Sci. Tech..

[40] Agüero M.B., González M., Lima B., Svetaz L., Sánchez M., Zacchino S., Feresin G., Schmeda-Hirschmann G., Palermo J., Wunderlin D. (2010). Argentinean propolis from *Zuccagnia punctata* Cav. (Caesalpinieae) exudates: Phytochemical characterization and antifungal activity. J. Agric. Food Chem..

[41] Vera N., Solórzano E., Ordóñez R., Maldonado L., Bedascarrasbure E., Isla M.I. (2011). Chemical composition of Argentinean propolis collected in extreme regions and its relation with antimicrobial and antioxidant activities. Nat. Prod. Commun..

[42] Danert F.C., Zampini C., Ordoñez R., Maldonado L., Bedascarrasbure E., Isla M.I. (2014). Argentinean Propolis as non conventional functional foods. Nutritional and functional composition. Nat. Prod. Commun..

[43] Salas A.L., Ordóñez R.M., Silva C., Maldonado L., Bedascarrasbure E., Isla M.I., Zampini I.C. (2014). Antimicrobial activity of Argentinean propolis against *Staphylococcus* isolated of canine otitis. J. Exp. Biol. Agric. Sci..

[44] Salas A.L., Alberto M.R., Zampini I.C., Cuello A.S., Maldonado L., Ríos J.L., Isla M.I. (2016). Biological activities of polyphenols-enriched propolis from Argentina arid regions. Phytomedicine.

[45] Solórzano E., Vera N., Cuello S., Ordóñez R., Zampini C., Maldonado L., Bedascarrasbure E., Isla M.I. (2012). Chalcones in bioactive Argentine propolis collected in arid environments. Nat. Prod. Commun..

[46] Solórzano E.R., Bortolini C., Bogialli S., Di Gangi I.M., Favaro G., Maldonado L., Pastore P. (2017). Use of a LC-DAD-QTOF system for the characterization of the phenolic profile of the argentinean plant *Zuccagnia punctata* and of the related propolis: New biomarkers. J. Func. Foods.

[47] Solorzano E.R., Di Gangi I.M., Roverso M., Favaro G., Bogialli S., Pastore P. (2019). Low level of allergens in the Argentinean plant *Zuccagnia punctata* Cav.: Screening and Quality Control of North-Western Propolis Using an LC-DAD-QTOF System. Appl. Sci..

[48] González M., García M.E., Slanis A., Bonini A., Fiedler S., Fariña L., Dellacassa E., Condurso C., Lorenzo D., Russo M. (2019). Phytochemical findings evidencing botanical origin of new propolis type from north-west Argentina. Chem. Biodivers..

[49] Lima B., Tapia A., Luna. L., Fabani M.P., Schmeda-Hirschmann G., Podio N.S., Wunderlin D.A., Feresin G.E. (2009). Flavonoids, DPPH activity, and metal content allow determination of the geographical origin of propolis from the Province of San Juan (Argentina). J. Agric. Food Chem..

[50] Lozina L., Peichoto M., Acosta O., Granero G. (2010). Standarization and organoleptic and physicochemical characterization of 15 Argentinean Propolis. Lat. Am. J. Pharm..

[51] Isla M.I., Carrasco Juárez B., Nieva Moreno M.I., Zampini I., Ordóñez R., Sayago J., Cuello S., Alberto M.R., Bedescarrabure E., Alvarez A. (2009). Effect of seasonal variations and collection form on antioxidant activity of propolis from San Juan, Argentina. J. Med. Food.

[52] Kumazawa S., Ahn M.R., Fujimoto T., Kato M. (2010). Radical-scavenging activity and phenolic constituents of propolis from different regions of Argentina. Nat. Prod. Res..

[53] Tosi E.A., Re E., Ortega M.E., Cazzoli A.F. (2007). Food preservative based on propolis: Bacteriostatic activity of propolis polyphenols and flavonoids upon *Escherichia Coli*. Food Chem..

[54] Busch V.M., Pereyra-Gonzalez A., Segatin N., Santagapita P.R., Ulrih N.P., Buera M.P. (2017). Propolis encapsulation by spray drying: 1 characterization and stability. LWT.

[55] Agüero M.B., Svetaz L., Sánchez M., Luna L., Lima B., López M.L., Zacchino S., Palermo J., Wunderlin D., Feresin G.E. (2011). Argentinean Andean propolis associated with the medicinal plant *Larrea nitida* Cav. (Zygophyllaceae). HPLC-MS and GC-MS characterization and antifungal activity. Food Chem. Toxicol..

[56] Mercado M.I., Moreno M.A., Ruiz A.I., Rodríguez I.F., Zampini C.I., Isla M.I., Ponessa G.I. (2018). Morphoanatomical and histochemical characterization of *Larrea* species from Northwestern Argentina. Rev. Bras. Farmacogn..

[57] Salas A., Mercado M.I., Zampini I.C., Ponessa G.I., Isla M.I. (2016). Determination of botanical origin of propolis from Monte region of Argentina by histological and chemical methods. Nat. Prod. Commun..

[58] Salas A.S., Mercado M.I., Orqueda E., Correa Uriburu F., García M.E., Pérez J., Alvarez M., Ponessa G., Maldonado L., Zampini I.C. (2020). *Zuccagnia*-type Propolis from Argentina: A potential functional ingredient in food to pathologies associated to metabolic syndrome and oxidative stress. J. Food Sci..

[59] Lersten N.R., Curtis J.D. (1996). Survey of leaf anatomy, especially secretory structures, of tribe Caesalpinieae (Leguminosae, Caesalpinioideae). Plant Syst. Evol..

[60] Mercado M.I., Ruiz A.I., Zampini I.C., Nuño G., Cuello S., Isla M.I., Ponessa G.I. (2013). Arquitectura y morfoanatomía foliar y caulinar de *Zuccagnia punctata* (Fabaceae). Histolocalización de compuestos bioactivos. Lilloa.

[61] Nieva Moreno M.I., Isla M.I., Cudmani N.G., Vattuone M.A., Sampietro A.R. (1999). Screening of antibacterial activity of Amaicha del Valle (Tucumán, Argentina) propolis. J. Ethnopharmacol..

[62] Salas A., Zampini I.C., Maldonado L., Isla M.I. (2018). Development of a bioproduct for medicinal use with extracts of *Zuccagnia*-type propolis. Nat. Prod. Commun..

[63] Nieva Moreno M.I., Isla M.I., Vattuone M.A., Sampietro A.R. (2000). Comparison of the free radical-scavenging activity of propolis from several regions. J. Ethnopharmacol..

[64] Isla M.I., Nieva Moreno M.I., Vattuone M.A., Sampietro A.R. (2001). Antioxidant activity of argentine propolis extracts. J. Etnopharmacol..

[65] Oldoni T.L., Cabral I., D’Arce M., Rosalen P., Ikegaki M., Nascimento A., Alencar S. (2011). Isolation and analysis of bioactive isoflavonoids and chalcone from a new type of Brazilian propolis. Sep. Purif. Technol..

[66] Tran V.H., Duke R., Abu-Mellal A., Duke C. (2012). Propolis with high flavonoid content collected by honey bees from Acacia paradoxa. Phytochemistry.

[67] Biharee A., Sharma A., Kumar A., Jaitak V. (2020). Antimicrobial flavonoids as a potential substitute for overcoming antimicrobial resistance. Fitoterapia.

[68] Cardoso R.L., Maboni F., Machado G., Alves S.H., de Vargas A.C. (2010). Antimicrobial activity of propolis extract against Staphylococcus coagulase positive and Malassezia pachydermatis of canine otitis. Vet. Microbiol..

[69] Nuño G., Alberto M., Zampini I., Cuello S., Ordoñez R., Sayago J., Baroni V., Wunderlin D., Isla M.I. (2014). The effect of *Zuccagnia punctata* Cav, an Argentina medicinal plant, on virulence factors from Candida species. Nat. Prod. Commun..

[70] Isla M.I., Moreno A., Nuño G., Carabajal A., Aberto M.R., Zampini I.C. (2016). Zuccagnia punctata Cav.: A review of its traditional uses, phytochemistry, pharmacology and toxicology. Nat. Prod. Commun..

[71] Tangarife-Castaño V., Correa-Royero J., Zapata-Londoño B., Durán C., Stanshenko E., Mesa-Arango A.C. (2011). Anti-Candida albicans activity, cytotoxicity and interaction with antifungal drugs of essential oils and extracts from aromatic and medicinal plants. Infection.

[72] Carabajal M.P.A., Isla M.I., Zampini I.C. (2017). Evaluation of antioxidant and antimutagenic activity of herbal teas from native plants used in traditional medicine in Argentina. South Afr. J. Bot..

[73] Carabajal M.P.A., Isla M.I., Borsarelli C.D., Zampini I.C. (2020). Influence of *in vitro* gastro-duodenal digestion on the antioxidant activity of single and mixed three “Jarilla” species infusions. J. Herb. Med..

[74] Carabajal M.P.A., Perea M.C., Isla M.I., Zampini I.C. (2020). The use of jarilla native plants in a Diaguita-Calchaquí indigenous community from northwestern Argentina: An ethnobotanical, phytochemical and biological approach. J. Ethnopharmacol..

[75] Moreno M.A., Gómez-Mascaraque L., Arias M., Zampini I.C., Sayago J.E., Pino Ramos L.L., Schmeda-Hirschmann G., López-Rubio A., Isla M.I. (2018). Electrosprayed chitosan microcapsules as delivery vehicles for vaginal phytoformulations. Carbohydr. Polym..

[76] Avila V., Bertolotti S.G., Criado S., Pappano N., Debattista N., García N.A. (2001). Antioxidant properties of natural flavonoids: Quenching and generation of singlet molecular oxygen. J. Food Sci. Technol..

[77] Morán Vieyra F., Boggetti H., Zampini I., Ordoñez R., Isla M., Alvarez R., De Rosso V., Mercadante A., Borsarelli C. (2009). Singlet oxygen quenching and radical scavenging capacities of structurally related flavonoids present in *Zuccagnia punctata* Cav. Free Rad. Res..

[78] Hwang S.H., Wecksler A.T., Wagner K., Hammock B.D. (2013). Rationally designed multitarget agents against inflammation and pain. Curr. Med. Chem..

[79] Lucas L., Russell A., Keast R. (2011). Molecular mechanisms of inflammation. Antiinflammatory benefits of virgin olive oil and the phenolic compound oleocanthal. Curr. Pharm. Des..

[80] Kim H., Son K., Chang H., Kang S. (2004). Anti-inflammatory plant flavonoids and cellular action mechanisms. J. Pharm. Sci..

[81] Yadav V.R., Prasad S., Sung B., Aggarwal B.B. (2011). The role of chalcones in suppression of NF-κB-mediated inflammation and cancer. Int. Immunopharmacol..

[82] Alberto M.R., Nieva Moreno M.I., Zampini I.C., Isla M.I. (2007). Anti-inflammatory activity of structurally related natural flavonoids. Bol. Latinoam. Caribe Plantas Med. Aromát..

[83] Alvarenga L., Cardozo L.F., Borges N.A., Chermut T.R., Ribeiro M., Leite M., Mafra D. (2020). To bee or not to bee? The bee extract propolis as a bioactive compound in the burden of lifestyle disease. Nutrition.

[84] Pastor-Villaescusa B., Sanchez Rodriguez E., Rangel-Huerta O. (2018). Polyphenols in obesity and metabolic syndrome. Obesity.

[85] Mahapatra D.K., Asati V., Bharti S.K. (2015). Chalcones and their therapeutic targets for the management of diabetes: Structural and pharmacological perspectives. Eur. J. Med. Chem..

[86] Mahapatra D.K., Bharti S.K. (2016). Therapeutic potential of chalcones as cardiovascular agents. Life Sci..

[87] Cai C.Y., Rao L., Rao Y., Guo J.X., Xiao Z.Z., Cao J.Y., Wang B. (2017). Analogues of xanthone-chalcones and bis-chalcones as α-glucosidase inhibitors and anti-diabetes candidates. Eur. J. Med. Chem..

[88] Bale A.T., Khan K.M., Salar U., Chigurupati S., Fasina T., Ali F., Perveen S. (2018). Chalcones and bis-chalcones: As potential α-amylase inhibitors; synthesis, *in vitro* screening, and molecular modelling studies. Bioorg. Chem..

[89] Roco J., Alarcon G., Medina M., Zampini C., Isla M.I., Jerez S. (2017). Beneficial effects of hydroalcoholic extract and flavonoids from *Zuccagnia punctata* in a rabbit model of vascular dysfunction induced by high cholesterol diet. Med. Chem. Res..

[90] Roco J., Zampini C., Isla M.I., Jerez S. (2018). Oral administration of *Zuccagnia punctata* extract improves lipid profile, reduces oxidative stress and normalizes vascular function in hypercholesterolemic rabbits. Phytomedicine.

[91] Ordóñez R., Zampini I.C., Nieva Moreno M.I., Isla M.I. (2011). Potential application of Argentine propolis to control some phytopathogenic bacteria. Microbiol. Res..

[92] Moreno A., Vallejo A.M., Ballester A.R., Zampini C., Isla M.I., Lopez-Rubio A., Fabra M.J. (2020). Antifungal edible coatings containing Argentinian propolis extract and their application in raspberries. Food Hydrocoll..

[93] Zampini I.C., Villena J., Salva S., Herrera M., Isla M.I., Alvarez S. (2012). Potentiality of standardized extract and isolated flavonoids from *Zuccagnia punctata* for the treatment of respiratory infections by *Streptococcus pneumoniae*: In vitro and in vivo studies. J. Ethnopharmacol..

[94] Khan S. (2017). Recent advances in role of propolis as natural additive in poultry nutrition. J. Apic. Sci..

[95] Bankova V., Popova M., Trusheva B. (2016). New emerging fields of application of propolis. Maced. J. Chem. Chem. Eng..

